# Angiotensin-Related Peptides and Their Role in Pain Regulation

**DOI:** 10.3390/biology12050755

**Published:** 2023-05-22

**Authors:** Wataru Nemoto, Ryota Yamagata, Osamu Nakagawasai, Koichi Tan-No

**Affiliations:** Division of Pharmacology, Faculty of Pharmaceutical Sciences, Tohoku Medical and Pharmaceutical University, Sendai 981-8558, Japan; yamagata@tohoku-mpu.ac.jp (R.Y.); osamun@tohoku-mpu.ac.jp (O.N.); koichi@tohoku-mpu.ac.jp (K.T.-N.)

**Keywords:** angiotensin-converting enzyme, angiotensin II, angiotensin (1–7), AT1 receptor, AT2 receptor, MAS1 receptor, neuropathic pain, inflammatory pain

## Abstract

**Simple Summary:**

Evidence indicates that angiotensin (Ang)-related peptides, also known as hypertensive peptides, are involved in pain regulation. Ang-related peptides exhibit various physiological effects via their receptors expressed throughout the body, and changes in the expression of Ang system components at the site of a lesion affect the local inflammatory response and pain transmission. Therefore, an extensive understanding of the pain modulatory mechanisms of Ang-related peptides and their receptors in several tissues and organs will aid in the development of drug therapies targeting the Ang system. This review article focuses on the current evidence regarding the mechanisms of pain regulation by Ang-related peptides in the central and peripheral regions involved in pain transmission.

**Abstract:**

Angiotensin (Ang)-generating system has been confirmed to play an important role in the regulation of fluid balance and blood pressure and is essential for the maintenance of biological functions. Ang-related peptides and their receptors are found throughout the body and exhibit diverse physiological effects. Accordingly, elucidating novel physiological roles of Ang-generating system has attracted considerable research attention worldwide. Ang-generating system consists of the classical Ang-converting enzyme (ACE)/Ang II/AT1 or AT2 receptor axis and the ACE2/Ang (1–7)/MAS1 receptor axis, which negatively regulates AT1 receptor-mediated responses. These Ang system components are expressed in various tissues and organs, forming a local Ang-generating system. Recent findings indicate that changes in the expression of Ang system components under pathological conditions are involved in the development of neuropathy, inflammation, and their associated pain. Here, we summarized the effects of changes in the Ang system on pain transmission in various organs and tissues involved in pain development process.

## 1. Introduction

The angiotensin (Ang) system has been extensively studied in the field of cardiovascular health because of its influence on fluid balance and blood pressure regulation. Inhibitors of renin and Ang-converting enzymes and antagonists of the AT1 receptor are already widely used in clinical practice to target this system. Ang-related drugs have also shown efficacy in diabetes and related kidney diseases because the Ang system is also involved in obesity, inflammation, and insulin resistance [1]. The Ang system is composed of various bioactive peptides and their receptors, which are described extensively in this review. These components are expressed in various tissues and organs throughout the body and are involved in several physiological reactions. Accordingly, elucidating novel physiological roles of the Ang system have attracted considerable research attention worldwide. Accumulating evidence suggests the involvement of the Ang system in pain regulation. The fact that the Ang system, which has been regarded as a cardiovascular hormonal system, is also involved in pain transmission is intriguing and indicates the potential for further utilization of drugs targeting the Ang system. In this review, we summarized the mechanisms by which Ang-related peptides and their receptors regulate pain in the central (brain and spinal cord) and peripheral (dorsal root ganglia (DRG), sciatic nerve, joint and sensory nerve ending) areas.

## 2. Biosynthetic Mechanism of Angiotensin-Related Peptides

**Figure 1:**
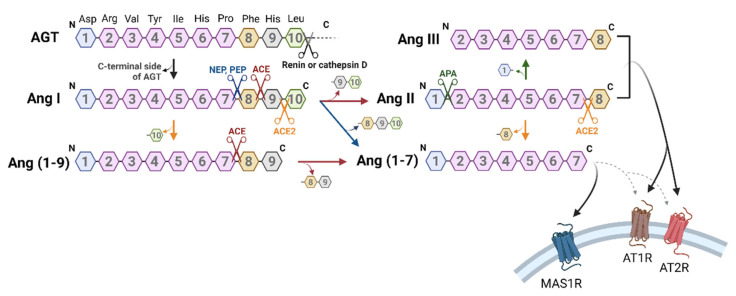
Biosynthetic mechanism of Ang-related peptides and their receptors. The ten N-terminal amino acids of AGT were sequentially numbered from the terminus Asp. Enzymes are represented in the form of scissors, with different colors for each type. The reaction catalyzed by each enzyme is indicated by the same-colored arrow. Abbreviations: ACE; angiotensin-converting enzyme; AGT, angiotensinogen; Ang, angiotensin; AT1R, Ang type 1 receptor; AT2R, Ang type 2 receptor; APA, aminopeptidase A; MAS1R, MAS1 receptor; NEP, neutral endopeptidase; PEP, prolyl endopeptidase.

The Ang-generating system consists of a cascade starting from angiotensinogen (AGT), which is acted on by degradative enzymes in a stepwise manner to produce various bioactive peptides (Figure 1). Ang II, the main bioactive peptide in the Ang-generating system, is thought to be produced in the circulating blood. Specifically, AGT produced in the liver is degraded by renin secreted from the kidney, and the resulting Ang I acts on the Ang-converting enzyme (ACE) expressed on vascular endothelial cells in the lung to produce Ang II. These Ang-generating system components are also found locally in various sites, including kidney [2], liver [3], brain [4], adipose tissue [5], DRG [6], spinal cord [7] and joint [8], constituting a local Ang-generating system. Ang II and III are agonists of Ang II type (AT) 1 and AT2 receptors, while Ang (1–7), the N-terminal fragment of Ang II, is known to act on the MAS1 receptors (Figure 1). Ang (1–7) has been reported to have opposing effects on various AT1 receptor-mediated physiological effects when bound to the MAS1 receptor [9], although several studies have suggested that it also binds to AT1 [10,11] and AT2 [12,13] receptors. Overall, the angiotensin-generating system is composed of the classical ACE/Ang II/AT1 receptor axis and the ACE2/Ang (1–7)/MAS1 receptor axis that negatively regulates it, and physiological functions are maintained by the mutual regulation of the balance between these two receptor systems.

## 3. Mechanisms of Pain Regulation in the Central Nervous System by Angiotensin-Related Peptides

### 3.1. Brain

**Figure 2:**
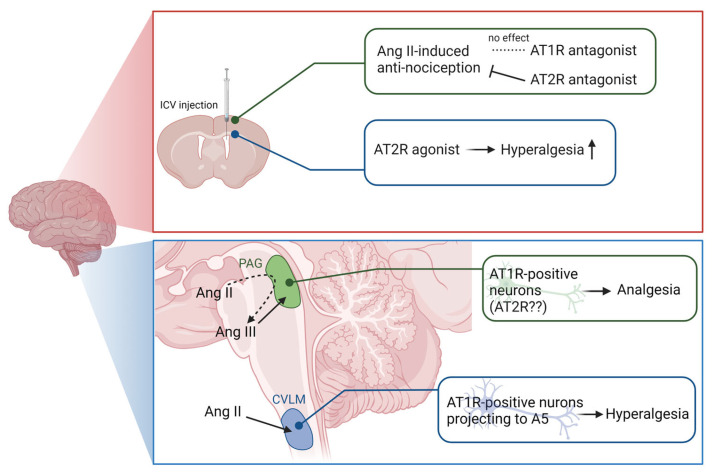
Pain regulation by Ang-related peptides and their receptors in the brain. Based on the results obtained by local administration of Ang-related peptides and ligands for their receptors in the brain, the involvement of the Ang system in the pain response is illustrated. Abbreviations: Ang, angiotensin; AT1R, Ang type 1 receptor; AT2R, Ang type 2 receptor; CVLM, caudal ventrolateral medulla; ICV, intracerebroventricular; PAG, periaqueductal gray; STZ, streptozotocin.

Ang-related peptide-mediated AT1 receptor activation is known to have opposing effects on pain depending on the brain region. For instance, microinjection of Ang II into the caudal ventrolateral medulla (CVLM), a supraspinal region that regulates pain, induces hyperalgesia via AT1 receptors [14]. In the CVLM, only 3% of AT1 receptor-positive neurons projected into the spinal cord, indicating that this pain response was not a direct neural pathway between the CVLM and spinal cord. Further studies showed that AT1 receptor-positive neurons in the CVLM project into the pontine noradrenergic A5 nucleus, and that activation of this neural circuit mediates pain signaling by Ang II [15]. Although studies are yet to confirm whether the CVLM-A5 pathway is activated by endogenous Ang II in animal models of pain, these findings indicate that Ang II may act as a pain modulator in this region (Figure 2).

In contrast, Ang II and III have been shown to act as analgesics when administered to periaqueductal gray matter (PAG) and can suppress plantar incision-induced allodynia [16,17]. Interestingly, the analgesic effect of Ang II was abolished by the aminopeptidase inhibitor amastatin [17]. Moreover, when incubated with PAG tissue homogenate, Ang II was degraded to Ang III, Ang (1–7) and Ang IV [17], indicating that several Ang system enzymes are expressed in this region. Since Ang II is metabolized to Ang III via APA (Figure 1), the active substance responsible for the analgesic action in PAG is thought to be Ang III. Although AT1 and AT2 receptors are expressed in the PAG [16], the exact receptor targeted by Ang III is unknown. Since the analgesic effects of Ang II and III are suppressed by AT1 receptor and AT2 receptor antagonist losartan and CGP42,112A, respectively, it could be concluded that both receptors are involved. However, it has been suggested that CGP42,112A may be a full agonist of the AT2 receptor [18], indicating the need for further studies on the specific role of AT2. Since losartan exacerbated incision-induced allodynia even when administered alone [16], it could be concluded that endogenous local Ang-producing system works in the analgesic direction in PAG via AT1 receptors (Figure 2).

Additionally, intracerebroventricular (ICV) administration of AT2 ligands facilitated the identification of several mechanisms of pain modulation in the brain via AT2 receptors. Intraperitoneal (IP) administration of 1% acetic acid solution induced a writhing response in mice owing to the contraction of abdominal smooth muscle; however, ICV administration of Ang II attenuated this response [19]. The anti-nociceptive effect of Ang II is AT2 receptor-dependent, as it was suppressed by the AT2 receptor antagonist PD123319 but not by losartan. Additionally, AT2 receptor-deficient mice had a lower pain threshold than wild-type mice, and the amount of β-endorphin in the arcuate nucleus of the hypothalamus, where the cell bodies of β-endorphin-containing nerves are located [20], was lower in AT2-deficient mice [21]. Moreover, ICV administration of the hexapeptide AT2 receptor agonist novokinin worsened streptozotocin (STZ)-induced hyperalgesia in type 1 diabetic rats [22]. Overall, these findings indicate that the administration of AT2 receptor ligands via the ICV route promoted pain exacerbation and suppression by agonists and antagonists, respectively (Figure 2).

### 3.2. Spinal Cord

**Figure 3:**
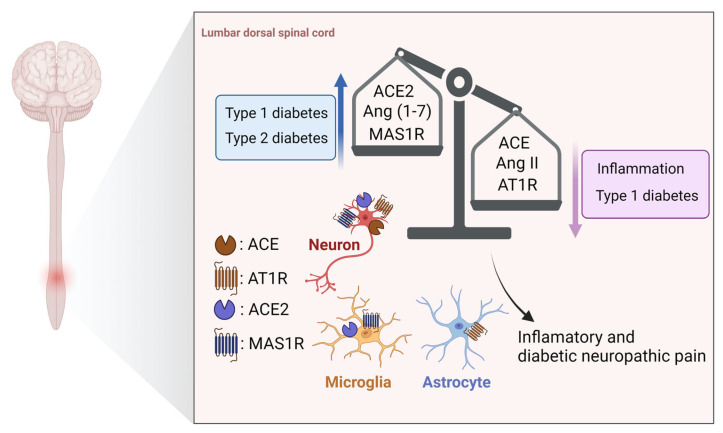
Pain regulation by Ang-related peptides and their receptors in the spinal cord. An imbalance between the ACE/Ang II/AT1R axis and the ACE2/Ang (1–7)/MAS1R axis in the spinal cord is observed under inflammatory and diabetic neuropathic pain, which is responsible for the development of pain. The distribution of the components of both axes in the dorsal horn of the spinal cord is also illustrated. Abbreviations: ACE; angiotensin-converting enzyme; Ang, angiotensin; AT1R, Ang type 1 receptor; AT2R, Ang type 2 receptor.

The expression of Ang II in the spinal cord was confirmed in the 1970s [23]. The dorsal spinal cord expresses all the components required for the construction of the ACE/Ang II/AT1 receptor axis and the ACE2/Ang (1–7)/MAS1 receptor axis except renin [7,24]. However, it is likely that the local Ang-generating system plays a role in pain regulation mechanisms in this region, based on the increased expression of Ang II in the spinal cord of animal models of inflammatory pain [25] and type 1 diabetes-induced neuropathic pain [7]. The expression patterns of various Ang-producing system components in spinal dorsal horn glial cells and neurons are summarized in Figure 3 [7,26,27,28]. The production of Ang-related peptides in the spinal cord requires other enzymes to fulfill the role of renin due to its absence in the spinal cord. One potential candidate enzyme is cathepsin D, which is structurally homologous to renin [29] and possess the ability to cleave AGT to Ang I [30] (Figure 1). Interestingly, cathepsin D has been found to be expressed in the dorsal spinal cord [7,31]. Moreover, receptor binding experiments using the AT1 receptor antagonist losartan and the AT2 receptor antagonist PD123319 [32] showed that AT1 receptors were highly expressed in the dorsal horn of the spinal cord, whereas AT2 receptors were undetected. Similarly, microphotometry using a specific antibody confirmed the distribution of AT1 receptor expression in the spinal cord [33]. Administration of AT1 receptor antagonist and not the AT2 receptor antagonist PD123319 suppressed intrathecal administration of Ang II or Ang III-induced pain-related behaviors (scratching, licking, biting; lasted for 30 min), indicating that these behaviors were AT1 receptor dependent. Additionally, the pain-related behaviors were linked with p38 MAPK activation in neurons and astrocytes, which are spinal AT1 receptor-positive cells [26,33]. However, the time course experiments measured at 2 h intervals after intrathecal administration of Ang II did not induce hyperalgesia [34].

Ang III administration has been shown to have a more potent pain-producing effect than Ang II [26]. This difference in intensity of action may involve the analgesic peptide Ang (1–7), which is produced from Ang II. Specifically, both Ang II and III are full agonists of the AT1 receptor, but Ang II, whose metabolite includes the analgesic peptide Ang (1–7), may have a weaker onset of pain than Ang III. Indeed, intrathecal administration of Ang (1–7) has been found to inhibit p38 MAPK activation and subsequent pain-related behaviors by Ang II and III via MAS1 receptors [24,28]. However, it is necessary to confirm whether the endogenous Ang (1–7)-generating system is involved in pain regulation. Previous studies have shown that the ACE/Ang II/AT1 receptor system was activated in the spinal cord of a mouse model of type 1 diabetes, whereas the ACE2/Ang (1–7)/MAS1 receptor system was dysfunctional [7,35] (Figure 3). Additionally, intrathecal administration of the AT1 receptor antagonist losartan and Ang (1–7) suppressed diabetic hyperalgesia, suggesting that an imbalance between these two Ang axes is involved in the onset of pain. Moreover, although Ang II-generating system was not activated in the spinal dorsal horn of leptin-deficient *ob/ob* mice, a model of type 2 diabetes, hyperalgesia was induced due to decreased ACE2 expression [36] (Figure 3). Overall, the spinal ACE2/Ang (1–7)/MAS1 receptor axis appears to contribute to the development of pain even when it is dysfunctional. Interestingly, Ang (1–7) also exhibits inhibitory effects on nociceptive behavior induced by the pain-producing substances NMDA and substance P (SP) [37]. In contrast, there were no functional changes in the spinal Ang (1–7)-generating system in mice with formalin-induced inflammatory pain, but activation of this system had anti-nociceptive effects [27] (Figure 3). Thus, the analgesic effects of Ang (1–7) may result not only from inhibition of the Ang II system but also from inhibition of a wide range of pain signals.

## 4. Mechanisms of Pain Regulation in Peripheral Tissue by Ang-Related Peptides

### 4.1. Dorsal Root Ganglia

A local Ang-generating system also exists in the DRG, and Ang II coexists with pain transmitters such as calcitonin gene-related peptide (CGRP) and SP [6]. The role of the Ang-system in the DRG has been extensively studied using a model of chemotherapy (anticancer drugs)-induced peripheral neuropathy (CIPN). CIPN is a frequently observed side effect in cancer patients, and an aggregate analysis of 4179 patients in 31 studies reported CIPN prevalence of 68.1% at 1 month and 30% at 6 months after chemotherapy [38]. In addition to reducing a patient’s quality of life, CIPN can suppress the efficacy of chemotherapy. Recently, a preliminary cohort study of patients on platinum-based regimens reported significantly less sensory neuropathy after chemotherapy in patients taking Ang-related drugs (AT1 receptor blockers or ACE inhibitors) compared with the control group [39], indicating that the neuroprotective effects of Ang-related drugs may be effective in CIPN. Additionally, continuous administration of losartan inhibited paclitaxel-induced mechanical hyperalgesia in rats by decreasing the expression of interleukin (IL)-1β, tumor necrosis factor (TNF)-α and monocyte chemotactic protein (MCP)-1 in DRG [40]. Interestingly, the anti-CIPN effect of losartan was observed regardless of whether the treatment commenced before or after the onset of CIPN, indicating that inhibition of AT1 receptors in the DRG was effective in the prevention and treatment. Macrophages have been shown to secrete several cytokines in the DRGs during CIPN. For instance, the expression of the M1 macrophage markers C-C motif chemokine ligand 2 (CCL2), CD68, TNF-α, and IL-6 was elevated in the DRG in paclitaxel-treated CIPN rats; however, losartan treatment suppressed the expression of some of these markers and increased the expression of M2 macrophage markers, including arginase-1 and IL-10, to exert anti-hyperalgesic effects [41]. Additionally, satellite glial activation was also suppressed by losartan. Administration of minute amounts of Ang II (subpressor doses) that are capable of exacerbating chronic constriction injury (CCI)-induced neuropathic pain via AT1 receptors, increased the activation of DRG neurons via increased satellite glial activity [42].

Similar to the spinal cord, components of the Ang (1–7)-generating system, such as ACE2 [43,44] and MAS1 receptors [35,45], are expressed in the DRG. A previous study showed a daily increase in the expression of MAS1 receptors in the DRG of rat with neuropathic pain after surgery, and that exogenous Ang (1–7) administration had a marked anti-hyperalgesic effect [45]. However, the effect of Ang (1–7) was abolished by the MAS1 receptor antagonist A779, indicating that the anti-hyperalgesic effect was MAS1 receptor-dependent. In contrast, although neuropathic pain was observed in STZ mice, a model of type 1 diabetes, MAS1 receptor expression in the DRG was unaffected [35].

Regarding AT2 receptors, although they are not expressed in the DRGs, it has been demonstrated that AT2 receptor-positive macrophages activated at the site of injury play an important role in peripheral pain signaling [34,46]. For example, activated AT2 receptor-positive macrophages at the site of injury activated transient receptor potential ankyrin 1 (TRPA1) in the DRG via release of reactive oxygen species, inducing hyperalgesia in mouse model of neuropathic pain [46]. Several mechanisms of pain control via regulation of DRG neural activity by AT2 receptors have been reported using AT2 receptor antagonists and AT2 receptor-deficient mice; however, it is presumed that the mechanism is probably via AT2 receptors on macrophages in the periphery. For instance, mice treated with resiniferatoxin (RTX), a potent transient receptor potential vanilloid 1 activator, showed hypoalgesia due to reduction of CGRP and SP in the intraepidermal nerve and DRG; however, the hypoalgesic effect was suppressed by continuous administration of the AT1 receptor antagonist candesartan from a day before RTX administration [47]. Since the effect of candesartan was abolished by the AT2 receptor antagonist EMA200 and in AT2 knockout mice, the neuroprotective effect could be attributed to indirect AT2 receptor activation by AT1 blockade rather than via AT1-dependent effect. In contrast, recent findings suggest that the inhibition of AT2 receptor signaling is effective against hyperalgesia. For example, the ACE inhibitor ramipril was effective against paclitaxel-induced neuropathic pain by decreasing DRG nerve deletion; however, this anti-hyperalgesic effect was abolished in AT2 receptor-deficient mice [48]. Moreover, the activation of AT2 receptors has been implicated in the development of CCI-induced neuropathic pain [49], prostate cancer-induced bone pain [50] and vestibular neuralgia [51] via modulation of neural activity in the DRG. Overall, these findings indicate that the AT2 receptor is a target for pain treatment. Notably, large-scale clinical trials have been conducted to examine the mechanism of EMA401, an AT2 receptor antagonist. A multicenter, randomized, double-blind phase II clinical study of 183 patients with postherpetic neuralgia, demonstrated that EMA401 induced significant analgesic effects [52]. Moreover, a subsequent clinical trial in patients with painful diabetic neuropathy in addition to postherpetic neuralgia confirmed the analgesic potential of EMA401; however, the trial was discontinued after a parallel preclinical study found that EMA401 could cause severe hepatotoxicity [53].

**Figure 4:**
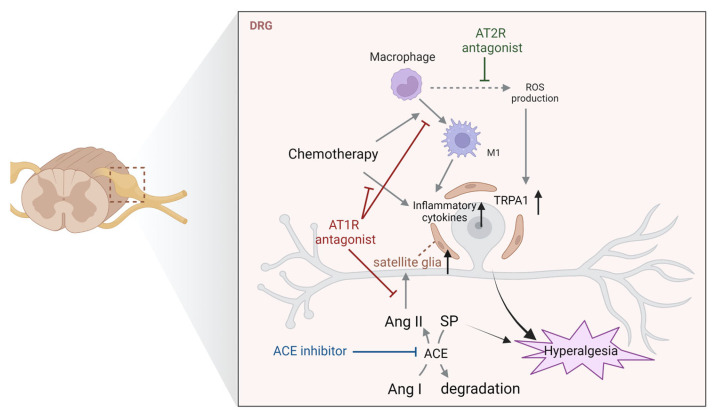
Pain regulation by Ang-related peptides and their receptors in the DRG. Schematic of previously reported mechanisms of pain development via the Ang system in the DRG. Abbreviations: ACE; angiotensin-converting enzyme; Ang, angiotensin; AT1R, Ang type 1 receptor; AT2R, Ang type 2 receptor; ROS, reactive oxygen species; SP, substance P; TRPA1; transient receptor potential ankyrin 1.

In contrast, a previous study reported that ACE inhibitors do not necessarily suppress pain. Specifically, the ACE inhibitors captopril and enalapril induced mechanical hyperalgesia in naive mice when administered IP or intrathecally [54], which was attributed to an increased expression of SP in the DRG and dorsal horn of the spinal cord. However, the pain was inhibited by antagonist of the NK1 receptors, one of the receptors for SP, or exogenous ACE administration. SP is degraded by several enzymes, including ACE [55], neutral endopeptidase [56], SP-degrading enzyme [55], post-proline cleaving enzyme [57], dipeptidyl aminopeptidase IV [58], cathepsin D [59], aspartic endopeptidase [59], and cathepsin E [60]. Inhibition of one of these enzymes, appears to result in the accumulation of endogenous SP in the DRG. ACE inhibitor-induced increase in SP has also been observed in rat brain after traumatic brain injury [61], indicating that ACE plays an important role in SP degradation. Overall, ACE inhibitors have been reported to have a bidirectional action of inducing hyperalgesia in the DRG via an increase in the pain-producing peptide SP, as well as a neuroprotective action. Based on these findings, the pain regulatory mechanisms via the Ang system in the DRG are summarized in Figure 4.

### 4.2. Sciatic Nerve

**Figure 5:**
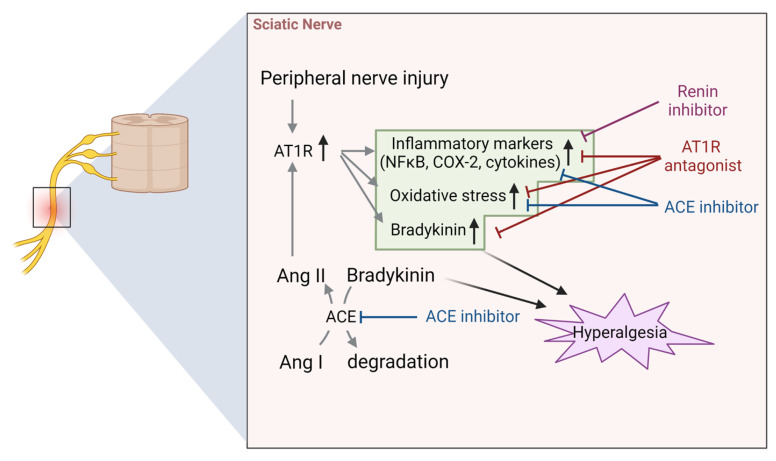
Pain regulation by Ang-related peptides and their receptors in the sciatic nerve. Schematic of the previously reported mechanisms of pain development via the Ang system in sciatic nerve. Abbreviations: ACE; angiotensin-converting enzyme; Ang, angiotensin; AT1R, Ang type 1 receptor; COX-2, cyclooxygenase-2; NFκB, nuclear factor κB.

AT1 receptors are also expressed in the sciatic nerve, and their expression has been reported to increase at the site of injury [32,62]. Interestingly, AT1 receptors are upregulated upstream (closer to the DRG) than peripherally when ligating two nearby sites on the sciatic nerve, suggesting that the change in receptor abundance reflects axonal transport rather than local biosynthesis. Experiments using retrograde tracers have shown that DRGs may be a source of AT1 receptors in the sciatic nerve [62]. Several studies have been performed on the mechanisms of pain regulation via the ACE/Ang II/AT1 receptor axis in the sciatic nerve. For instance, treatment with the AT1 receptor antagonist telmisartan for two consecutive weeks suppressed CCI-induced increase in TNF-α expression in sciatic nerve in a rat model of sciatic nerve ligation, thereby ameliorating thermal and mechanical hyperalgesia [63] (Figure 5). Similarly, treatment with aliskiren, a direct inhibitor of the Ang I synthase renin, suppressed CCI-induced hyperalgesia via inhibition of TNF-α in the sciatic nerve [64] (Figure 5).

Ang-related drugs work on the peripheral nervous system as well as the brainstem, which is involved in the maintenance of neuropathic pain [65,66]. Overall, AT1 receptor antagonists and ACE inhibitors are classified into two categories: those that are centrally-transferable (cross the blood-brain barrier) and those that are not. Hegazy et al. reported that in a rat model of CCI-induced neuropathic pain, AT1 receptor antagonists and ACE inhibitors had similar neuroprotective and analgesic effects in both the central-transferable drug group (telmisartan [67] and ramipril [68]) and the peripheral-acting drug group (losartan and enalapril [69]) [70]. Interestingly, AT1 receptor antagonists showed anti-hyperalgesic effects with suppression of inflammatory markers (NFκB, TNF-α, cyclo-oxygenase-2), oxidative stress markers (NADPH oxidase and catalase), and serum bradykinin levels in the sciatic nerve and brain stem; in contrast, ACE inhibitors did not suppress bradykinin levels. Thus, while AT1 receptor antagonists and ACE inhibitors exhibit neuroprotective effects in CCI model, they show differences in action with respect to bradykinin, one of the biochemical markers that is facilitative to pain (Figure 5).

Furthermore, AT1 receptor antagonists have been reported to act in the analgesic direction, whereas ACE inhibitors act in both directions (analgesia or hyperalgesia). Specifically, ACE inhibitors exacerbate pain via activation of bradykinin signaling in the sciatic nerve in CIPN models [71,72]. Similar to SP in the DRG, bradykinin is a substrate for ACE, and ACE inhibitors inhibit the degradation of bradykinin [73,74] (Figure 5). Bradykinin promotes pain resulting from vincristine- [75] and paclitaxel-induced [76] CIPN via activation of its own receptors, the B1 and B2 receptors. Increased expression of B1 and B2 receptors was observed in the sciatic nerve of a mouse model of paclitaxel-induced neuropathic pain, forming a lesion that is prone to bradykinin action; additionally, the ACE inhibitor enalapril enhanced hyperalgesia via activation of these receptors [71]. Although another study confirmed that the ACE inhibitors captopril and enalapril exacerbated paclitaxel-induced neuropathic pain, the AT2 receptor antagonists PD123319 and EMA401 suppressed it, while the AT1 receptor antagonist losartan was ineffective against the pain [72]. However, this study examined the acute effects of losartan, which differs in duration of administration from the above findings that AT1 receptor antagonists were neuroprotective. Although the reasons for the bidirectional effects of ACE inhibitors are currently unknown, it has been reported that increased bradykinin levels are not always observed during ACE inhibitor treatment [77], indicating that it is possible that the different/opposing effects may be dependent on the subject’s condition and lesions in the area causing the pain.

### 4.3. Joint and Bone Tissues

**Figure 6:**
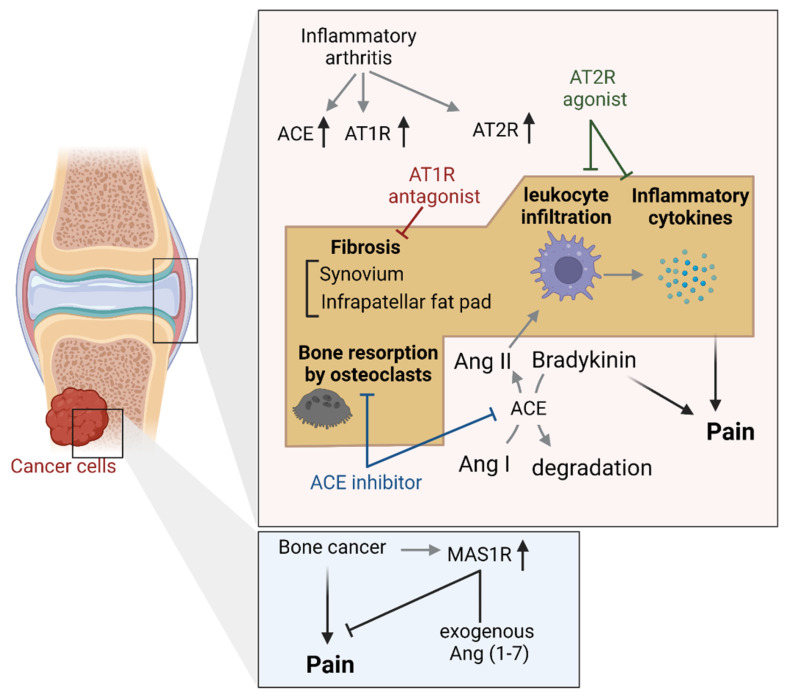
Pain regulation by Ang-related peptides and their receptors in the bone tissue. Schematic of previously reported mechanisms of pain development via the Ang system in joint and bone tissues. Abbreviations: ACE; angiotensin-converting enzyme; Ang, angiotensin; AT1R, Ang type 1 receptor; AT2R, Ang type 2 receptor; MAS1R, MAS1 receptor.

Increased expression of AT1 receptors have been reported in rat models of inflammatory arthritis [78] and synovial tissue in osteoarthritis (OA) patients [79]. Although cartilage deterioration is the primary factor in the development of OA, pain caused by OA has been shown to involve non-cartilaginous structures within the joint, including fibrosis of the infrapatellar fat pad (IFP) [80] and synovium [81]. The activation of AT1 receptors contributes to tissue fibrosis in a facilitative manner in various tissues, such as liver, kidney, heart and skin [82], and this mechanism is likely to also occur in joint tissues. Notably, mechanical hyperalgesia along with fibrosis of IFP and synovium was observed in rats with monoiodoacetic acid (MIA)-induced OA; however, oral administration of the AT1 receptor antagonist losartan for four weeks induced antifibrotic and anti-hyperalgesic effects [83] (Figure 6). Similarly, another study showed that in addition to fibrosis of the synovium, bone resorption by osteoclasts and infiltration of leukocytes into the inflammatory site were observed in a model of MIA-induced arthritis; however, these pathological changes were suppressed by the ACE inhibitor captopril [84] (Figure 6). Interestingly, intra-articular administration of Ang II can induce OA-like lesions and associated hyperalgesia, indicating that Ang II may act as an arthritogenic factor [84]. Additionally, elevated expression of ACE and AT1 and AT2 receptors have been observed in the synovium of rats with collagen-induced OA (CIA) and in human OA [78] (Figure 6). Moreover, accelerated bone resorption and decreased bone formation were simultaneously observed in CIA rats; however, bone loss due to these pathological changes was suppressed by the ACE inhibitor perindopril [78]. Although perindopril treatment has been shown to induce joint protection in CIA rats with increased ACE and AT1 and AT2 receptors, the specific receptor by which Ang II exacerbate arthritis in the joint region is unknown. Given the finding that the selective AT2 receptor agonists suppressed local inflammatory cytokines and monocyte migration in animal models of arthritis both in vivo and in vitro studies [85,86,87] (Figure 6), it could be speculated that Ang II produced in joints exacerbates inflammation via AT1 receptors. Moreover, local Ang II production has been implicated in lumbar intervertebral disc degeneration [88], which causes lower back pain, and pain associated with tibial fracture orthopedic injury [89].

Similar to findings in the sciatic nerve and DRG, ACE inhibitors have been reported to exert bidirectional effects on pain (analgesic and anti-analgesic effects) in the joint region. In contrast to the joint-protective effects reported in OA model, ACE inhibitors contribute to the onset of gout attacks in a facilitatory manner by lowering the threshold of monosodium urate crystals mass required for the onset of acute gout attacks in a bradykinin B1 receptor-dependent manner [90]. This is due to the fact that ACE inhibitors activate B1 receptors by blocking the breakdown of bradykinin; however, ACE inhibitors appear to work in the opposite direction in animal models of gout attacks, worsening pain (Figure 6).

Furthermore, Ang (1–7) has been reported to inhibit cancer-induced bone pain (CIBP) via the MAS1 receptor. In a mouse model of CIBP, which was generated by implanting cancer cells into the femur, Ang (1–7) exerted anti-nociceptive effects via MAS1 receptors in femur extrudate and the DRG without affecting tumor volume or bone metabolism [91] (Figure 6). Particularly, a marked increase in MAS1 receptor expression has been observed in the femur of CIBP mice, improving the efficacy of Ang (1–7) in the lesion site. Opioids are commonly used for cancer pain, but they also possess bone loss [92,93,94] and tumor growth promoting effects [95,96,97], both of which can interfere with cancer treatment and reduce the patient’s quality of life. Therefore, the development of non-opioid analgesics is desired, and it has been suggested that the Ang (1–7)/MAS1 receptor system may be a novel drug target to meet this need.

### 4.4. Other Findings in Peripheral Pain Models

In animal models of pain induced by intradermal administration of pain-producing substances such as complete Freund’s adjuvant (CFA) or prostaglandin E2 (PGE2), pain regulation mechanisms by angiotensin-related receptors and their ligands around the site of injection have been reported. The anti-nociceptive effects of Ang (1–7) in the periphery were first demonstrated in rat experiencing PGE2-induced pain [98]. In this study, PGE2-induced hyperalgesia was suppressed by intraplantar administration of Ang (1–7), demonstrating the analgesic effect of Ang (1–7) on nerve endings. However, the anti-nociceptive effect of Ang (1–7) was blocked by the MAS1 receptor antagonist A779, but was not affected by naloxone administration, indicating that it was caused in an opioid-independent manner via the MAS1 receptor. Subsequent behavioral study revealed that the anti-nociceptive effect of Ang (1–7) was due to the activation of the neuronal nitric oxide synthase/cyclic GMP pathway and ATP-sensitive K^+^ channels locally at the site of administration (plantar region) [99]. Overall, these findings suggest that the Ang (1–7) system in peripheral nerve endings could be a potential therapeutic target for pain.

In an intraplantar CFA-induced inflammation model, increased expression of renin and AGT, components of the Ang II-generating system, was observed in T cells and macrophages at the inflammation site, along with increased intradermal nerve fiber (IENF) density [100]. Intraperitoneal administration of the AT2 receptor antagonist PD123319 to this CFA model attenuated mechanical and thermal hyperalgesia and decreased IENF density, indicating the proinflammatory effect of AT2 receptors in the periphery. Similarly, PD123319 treatment decreased IENF density around the injection site and hyperalgesia in a rat model of vaginal pain [101]. These findings suggest the potential of AT2 receptor antagonists as therapeutic targets for pain. Several preclinical studies have identified AT2 receptors on immune cells as therapeutic targets for analgesia [46,49,100]; moreover, recent reports have shown that naloxone suppresses the analgesic effects of AT2 receptor antagonists in irritable bowel syndrome-induced pain [102], indicating that AT2 signaling can also interfere with the opioid pathway.

## 5. Conclusions

This review examined the mechanisms of Ang-related peptides and their receptors in pain regulation. Drugs that target the Ang system have been demonstrated to be useful in treating neuropathic and inflammatory pain by acting on Ang-related peptide receptors found throughout the body. Presently, therapeutic agents, such as AT1 receptor antagonists and renin and ACE inhibitors, are already being used for the treatment of several diseases globally, and have been confirmed to be sufficiently safe. In contrast, regarding the AT2 receptor, while clinical trials of EMA401 have been suspended due to the risk of severe liver damage, this receptor remains an important analgesic target. Moreover, although ligands targeting the MAS1 receptor system have not yet been used in clinical practice, they are gaining considerable research interest as novel target for pain treatment. The Ang system is expected to exert pain modulating effects through a novel mechanism, and further research is expected to be developed in the future.

## Data Availability

Not applicable.
